# Medical tourism: the role of the primary care provider

**DOI:** 10.3399/bjgpopen17X100617

**Published:** 2017-04-05

**Authors:** Jamie L Weis, R Barry Sirard, Patrick A Palmieri

**Affiliations:** 1 Physician Assistant and Medical Service Trip Leader, Urgent Care & Primary Care, Coordinated Health, Allentown, Pennsylvania, US; 2 Primary Care Physician, Urgent Care & Primary Care, Coordinated Health, Bethlehem, Pennsylvania, US; 3 Director, Institute for Health Services Research, Universidad Privada del Norte, Lima, Peru

**Keywords:** medical tourism, transplant tourism, travel medicine, organ trafficking, primary health care, family practice

With medical costs rising and millions uninsured or underinsured, patients are paying cash in developing nations to access deeply discounted medical procedures. While medical tourism can be a cost-effective option, the phenomenon is not without risk: communication difficulties, endemic tropical diseases, unregulated hospitals, and organ trafficking complicate the landscape. These risks are precisely what put the well-informed provider in a position to educate patients to safely engage in the process.

**Figure 1.**
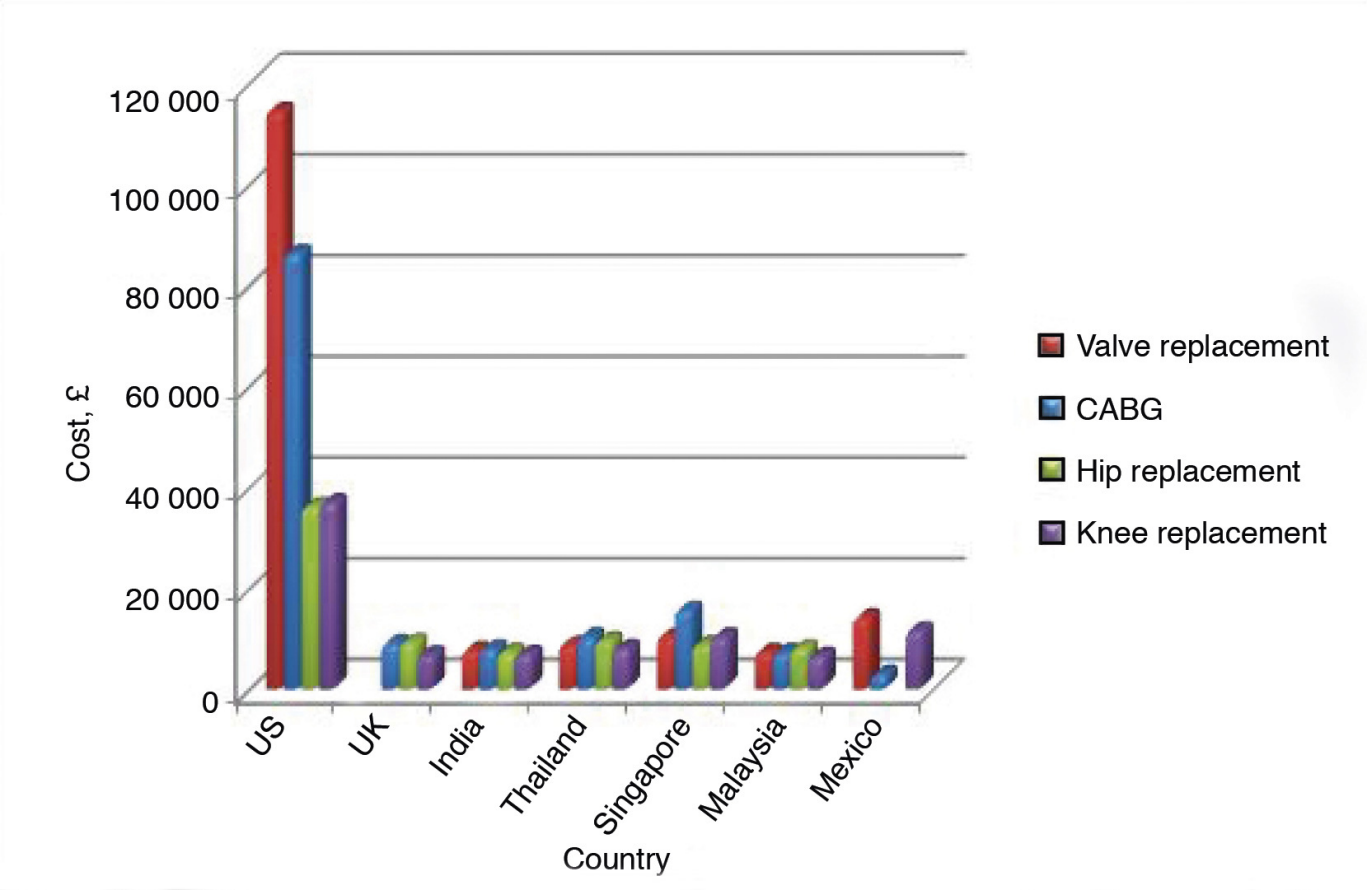
Comparative costs of medical procedures in different countries in US$.^4^ CABG = coronary artery bypass grafting.

The incidence is difficult to calculate. An estimated 50 million patients travel abroad each year seeking medical services and 3–20% of Europeans receive treatment in another European Union country.^1,2 ^Patients cite shorter waiting times, and lower costs as the primary motivators.^2^ Cardiac surgery or a knee replacement in a developing country can be a fraction of the cost due to currency exchange rates, lower labour costs, fewer regulations, little or no involvement of insurance companies, and low malpractice premiums.^3^ For example, a cardiac surgery averaging $113 000 (£85 880) in the US is done in India for only $10 000 (£7600).^4^ Figure 1 shows a cost comparison in GBP.^4^

Medical tourists usually fall into one of two categories: the middle-aged, middle-income uninsured or underinsured individuals who need a medical procedure; or middle-income individuals seeking an elective procedure.^3^ The common thread is that both have adequate resources to afford health care in developing nations, but neither can afford the same within their own local market.^3^

Some private insurers provide financial incentives for members to access procedures abroad.^5^ One company, Hannaford in conjunction with Aetna, offered substantial discounts to employees for joint replacements in Singapore, eventually enabling them to negotiate a lower cost with a US-based hospital.^5^

Patients cite word-of-mouth and internet searches as the leading determinants of destination.^6^ Patients then research online, despite little to no outcomes data, choosing among numerous medical tourism companies. These sites sell custom-made packages in warm climates, for the procedure as well as ancillary arrangements such as hotel, transfers, and flights.^1^

The most frequently sought treatments are cosmetic surgery, cardiac surgery, joint replacements, bariatric surgery, and organ transplant.^1^ Medical touring patients may have additional risks associated with communication and language barriers, counterfeit or low-quality medications, and bacterial infections with antibiotic resistance patterns different than in the home country.^7^

One analysis found a threefold greater postoperative infection rate in intensive care units outside Europe and the US.^7^ Many destinations also have increased risks of endemic diseases such as HIV, malaria, dengue fever, and West Nile virus. Joint commission-accredited hospitals screen blood transfusions for HIV and hepatitis B and C, but not for all regional vector-borne diseases and there have been at least 45 documented cases of malaria transmitted by organ transplant.^7^ In addition to infectious disease risk, one should also recognise the medical risk associated with air travel. As well as long flights increasing DVT risk, the change in altitude and pressure associated with commercial flights can cause postoperative complications with thoracic or abdominal surgery and the Aerospace Medical Association recommends waiting 1–2 weeks before flying after surgery.^8^

Other risks are more closely related to systems than to the procedure itself. If records are incomplete or not transferred entirely, particularly with medical complications, the continuity of care is hindered.^5^ Furthermore, many surgeons are apprehensive about accepting the potential complications and liability of caring for another surgeon’s patient.^5^

**Figure 2.**
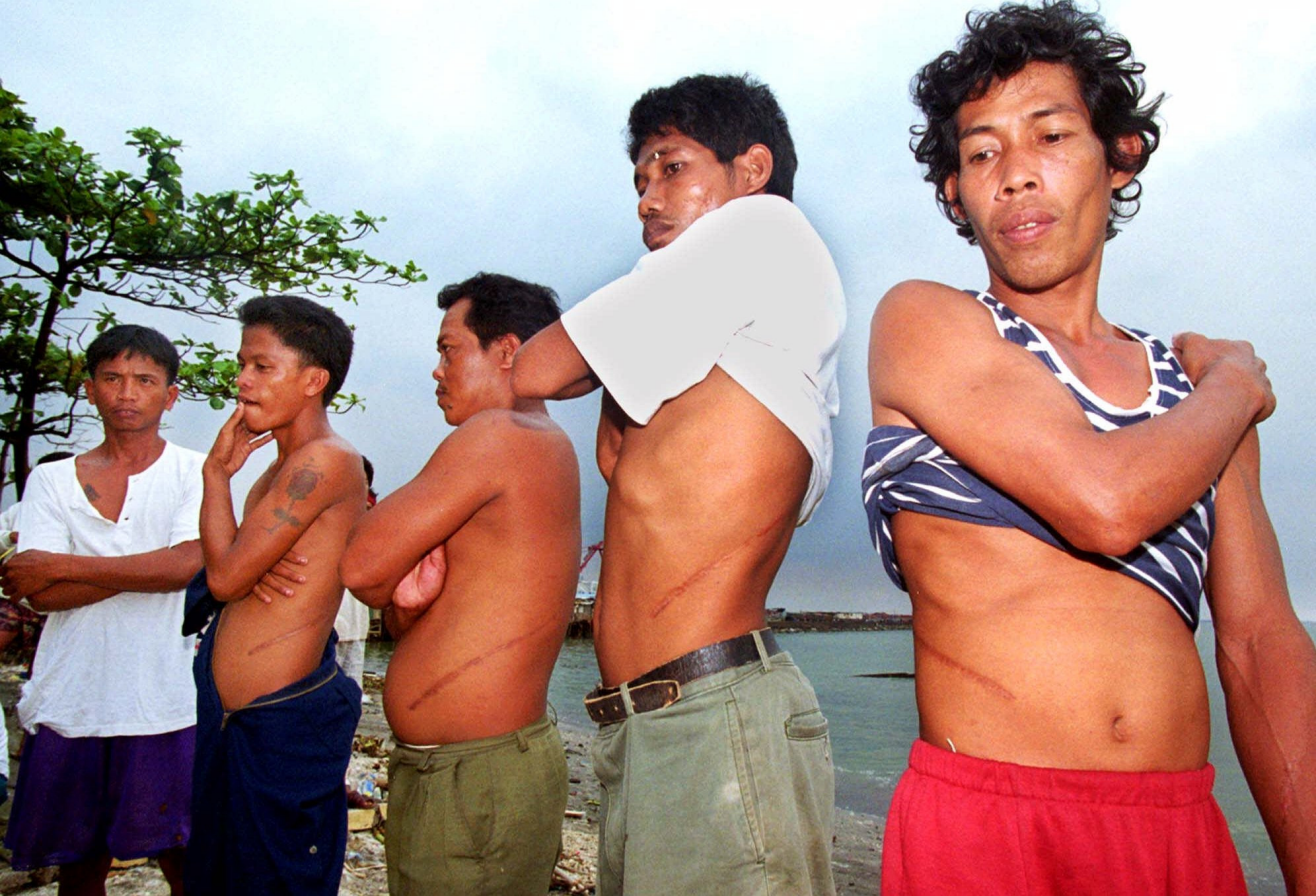
Filipino men displaying their scars from kidney surgery.^10^

Transplant tourism carries special risks including inadequate immunosuppression and a high rate of infection incidence^7,9^ including increased risk of developing hepatitis B and C, HIV, malaria, and tuberculosis compared to other forms of medical tourism.^7 ^Additionally, there are inequalities and ethical questions related to organ trafficking. The US Department of State 2015 *Trafficking in Persons Report* details how people, often impoverished young men, are convinced by traffickers to sell a kidney. Following this, they have poor medical treatment, and little to no follow-up care for complications.^9^ Figure 2 shows Filipino men displaying their scars from kidney sales.^10^

Unregulated, promotional medical tourism websites can present a skewed view of the true risks of surgery. Only 47% of these websites present any data on surgical risks.^11^ Online brokers, often paid by hospitals, will direct patients to the highest paying facilities typically without disclosing this conflict.^11^

To improve informed consent, providers can validate the destination hospital’s accreditation. QHA Trent, the organisation that accredits hospitals in the UK also accredits hospitals internationally. If a hospital outside the UK pursues and achieves accreditation to the QHA Trent standard, they will typically display the logo on their website.^12^ Similarly, Accreditation Canada International, Australian Counsel on Health Care Standards International, and Acreditas Global accredit international hospitals and ambulatory centers to a similar standard. The Joint Commission International (JCI) accredits international hospitals outside Australia, Canada, UK, and US, to a similar standard as the US and up-to-date listings appear on its website.^13^

Additional recommendations include seeing a travel medicine specialist 6 weeks before leaving, getting documentation on exactly what is covered, carrying medical records to and from the destination hospital, and making arrangements for postoperative follow-up. See Box 1.

**Box 1.** Recommendations for medical touring patients^8,12^

See a travel medicine specialist at least 6 weeks before leaving Get documentation on exactly what is covered by the cost Take all medical records with you on the trip Make arrangements for follow up with a physician when you get home Find out what activities (such as sun tanning or swimming) are safe after the operation Bring all surgical records back to the home physician Know whether the hospital is accredited and by whom Research thoroughly before considering organ transplant tourism Wait 1–2 weeks before flying after surgery

Providers who are willing to discuss the procedure openly, check accreditation, and recommend appropriate safety measures, can greatly improve the patient experience and ultimate outcome. As medical tourism continues to grow, it is important for providers to be aware of the trend and risks, and to assist in the education and informed consent of patients.
